# Exploring Factors Contribute to Adhering in Computerized Cognitive Training Among Healthy Older Adults in China

**DOI:** 10.1093/geroni/igab046.1872

**Published:** 2021-12-17

**Authors:** Yu Liu, Siman Lv, Cuiping Ni

**Affiliations:** 1 China Medical University School of Nursing, Shengyang, Liaoning, China (People's Republic); 2 China Medical University School of Nursing, Shenyang, Liaoning, China (People's Republic)

## Abstract

Computerized cognitive intervention has the potential to enhance cognition among healthy older adults. However, little is known of the factors associated with adherence in computerized cognitive training among healthy older adults in China. This study was designed to explore these factors utilizing a descriptive qualitative method. A semi-structured interview was used to interview 13 informants. The analysis suggested that factors associated with adherence to the computerized cognitive intervention, included 3 core themes:(1) individual characteristics, with three subthemes of “having free time”, “emotion”, and “persistence characteristics”; (2) encouragement, with three subthemes of “peer group support”, “support from healthcare professional”, and “supervision from facilitators”; and (3) self-recognized improvement related to training, with two subthemes of “better brain function” and “emotion improved”. The results revealed multi-factors promote adherence including personal and social aspects.

